# Prostate Carcinogenesis with Diabetes and Androgen-Deprivation-Therapy-Related Diabetes: An Update

**DOI:** 10.1155/2012/801610

**Published:** 2012-06-26

**Authors:** Noboru Hara

**Affiliations:** Division of Urology, Department of Regenerative and Transplant Medicine, and Division of Molecular Oncology, Department of Signal Transduction Research, Graduate School of Medical and Dental Sciences, Niigata University, Asahimachi 1, Niigata 951-8510, Japan

## Abstract

Prostate cancer and the androgen deprivation therapy (ADT) thereof are involved in diabetes in terms of diabetes-associated carcinogenesis and ADT-related metabolic disorder, respectively. The aim of this study is to systematically review relevant literature. About 218,000 men are estimated to be newly diagnosed with prostate cancer every year in the United States. Approximately 10% of them are still found with metastasis, and in addition to them, about 30% of patients with nonmetastatic prostate cancer recently experience ADT. Population-based studies have shown that dissimilar to other malignancies, type 2 diabetes is associated with a lower incidence of prostate cancer, whereas recent large cohort studies have reported the association of diabetes with advanced high-grade prostate cancer. Although the reason for the lower prevalence of prostate cancer among diabetic men remains unknown, the lower serum testosterone and PSA levels in them can account for the increased risk of advanced disease at diagnosis. Meanwhile, insulin resistance already appears in 25–60% of the patients 3 months after the introduction of ADT, and long-term ADT leads to a higher incidence of diabetes (reported hazard ratio of 1.28–1.44). Although the possible relevance of cytokines such as Il-6 and TNF-**α** to ADT-related diabetes has been suggested, its mechanism is poorly understood.

## 1. Introduction

Prostate cancer and the hormonal therapy thereof (androgen deprivation therapy, ADT) have been associated with diabetes in terms of diabetes-associated carcinogenesis [1] and ADT-related metabolic disorder [2], respectively. The present paper systematically introduces prostate carcinogenesis with diabetes and ADT-related diabetes/insulin resistance both in epidemiological and etiological approaches.

## 2. Search Method

PubMed and MEDLINE searches were performed for articles published between January 1991 and November 2011 based on the following key words for diabetes-associated prostate carcinogenesis: prostate cancer AND insulin resistance, hyperglycemia, cancer risk, and diabetes. Literature on ADT-related diabetes was searched using the following keywords: androgen deprivation therapy OR hormone therapy AND diabetes, insulin resistance, hyperglycemia, and metabolic syndrome. Relevant articles on growth hormone (GH)/insulin-like growth factor (IGF)-1 and androgen metabolism were searched with similar strategy. Except for studies concerning statistics, meta-analysis, or reanalysis, review articles were excluded. All full papers based on evidence level 1 and 2 and full papers on level 3 supporting them were downloaded via the library of our institution, provided from other institutions, or purchased, and relevant articles on experimental studies were obtained by similar methods.

## 3. General Statistics of Prostate Cancer

Prostate cancer is a common malignancy around the world, and in the United States, about 218,000 and 32,000 men are estimated to be newly diagnosed with and to die of prostate cancer every year, respectively [3]. Therapeutic options for prostate cancer are determined with informed-consent according to the disease-specific risk and patient's conditions such as age and comorbidities. Although the prostate-specific antigen (PSA) test has led to a stage migration with increased low- to intermediate-risk localized disease, about 10% of the patients are still found with metastatic disease at diagnosis [4]. Additionally, 20–35% of the patients are categorized as having locally advanced disease or localized high-risk cancer based on high histopathological grade (Gleason score of 8–10) or high PSA level (serum PSA higher than 20 ng/mL) [5, 6].

## 4. The Presence of Diabetes and the Incidence of Prostate Cancer

Large cohort studies have shown that diabetes is associated with a higher incidence of many malignancies including lung, gastric, colorectal, liver, and pancreatic cancer [7, 8]. Several molecular mechanisms have been suggested for their association, for example, insulin resistance leading to high cell proliferation by the activation of the phosphatidylinositol 3-kinase/Akt/mammalian target of rapamycin pathway, elevated leptin/adiponectin linking to impaired anticancer immunity, and upregulated inflammation/tumor necrosis factor-alpha (TNF-*α*) leading to cancer cell survival; they produce a complex network with many-to-many correspondence [9, 10]. Conversely, the mentioned cohort studies reported a lower prevalence of prostate cancer among men with type 2 diabetes compared with that in men without diabetes. Recently, Turner and colleagues reported that diabetes was associated with a reduced risk of prostate cancer (odds ratio = 0.78; 95% CI: 0.61–0.99) [11], and a study referring to the Swedish national database and nationwide Cancer Registry also showed a lower risk of prostate cancer in a total of 125,126 registered type 2 diabetes men [12]. Most recently, Atchison and associates reported that men with diabetes had a decreased risk of prostate cancer (RR = 0.89, 95% CI = 0.87–0.91) [7]. These results imply a specific relationship between diabetes and prostate cancer; however, it remains unknown why type 2 diabetes is associated with a lower incidence of prostate cancer. In a retrospective study enrolling 3,162 consecutive men who underwent prostate biopsy, Moses and associates showed that, though not significant, those with diabetes had higher odds of histologically more aggressive disease (Gleason score of 7 or higher) than those without diabetes (OR 1.31, 95% CI: 0.98–1.74; *P* = 0.07) [13]. In their study, diabetes also led to an increased risk of overall prostate cancer in the cohort (OR 1.26, 95% CI: 1.01–1.55; *P* = 0.04). It is suggested that the study design and cohort in the study by Moses et al. mainly comprising men with elevated PSA on prostate cancer screening possibly involves different patients' background.

It is known from the results of recent population-based cohort studies that men with type 2 diabetes show lower PSA levels than those without diabetes [14–16]. Considering evidence on reduced PSA levels in diabetic men, is exposure to PSA screening associated with a reduced risk of prostate cancer in men with diabetes? In a longitudinal observational study enrolling 4,511 men with newly diagnosed prostate cancer between 1986 and 2004, Kasper et al. demonstrated the increased risk of prostate cancer in diabetic men after PSA era compared with that in pre-PSA era, although the odds ratio still remained low after PSA era (0.86) [17]. In a recent population-based study conducted in Taiwan, 985,815 study subjects including 104,343 diabetes patients identified in 1997 were followed up between 1998 and 2009; the unadjusted and adjusted risk ratios in diabetes men for incident prostate cancer were 6.97 (5.34–9.10) *P* < 0.001 and 1.56 (1.19–2.04) *P* = 0.0013, respectively [18]. However, the rate of exposure to PSA screening in this population was unclear.

The relevance of lower PSA levels to the reduced risk of prostate cancer in men with diabetes is thus equivocal, but men with diabetes potentially have more advanced disease at diagnosis where their PSA level reaches a certain cut-off/threshold. Correspondingly, two recent large cohort studies reported the association of diabetes with high/poor-risk disease: more advanced clinical stage and higher Gleason sore [1]. In a cohort study, Li et al. reported that men with diabetes had a higher risk of advanced prostate cancer with a multivariate adjusted HR of 1.89 (95% CI: 1.02–3.50) in 230 men with prostate cancer newly identified among 22,458 Japanese men [19]. Although retrospective, several large studies have also reported the relationship between diabetes and high-grade prostate cancer [20, 21].

Accordingly, diabetes is associated with a lower PSA level in the general population and a higher incidence of poor-risk prostate cancer in the screening-based cohort or regional cancer registration. The latter can be explained by the frequent reduced testosterone levels in men with increased insulin resistance or type 2 diabetes [22] and is concordant with previous study results on prostate cancer biology; low testosterone environment *in vivo* is involved in high Gleason score [23–25], advanced disease stages [26], and a poor prognosis [27, 28]. All of these studies have suggested that the adaptation of cancer cells to low-testosterone milieu links to their high viability and malignant potential. Most recently, Botto and associates reported a high incidence of predominant Gleason pattern 4 (histologically high-grade pattern) in men with prostate cancer and low serum testosterone [29]. They performed a prospective study on 452 men who underwent radical prostatectomy; the final study group comprised 431 eligible patients. In surgical specimens, 132 patients (31%) had predominant Gleason pattern 4, and their serum total testosterone level was lower than that in the remaining 299 with predominant lower histological grade (4.00 versus 4.50 ng/mL, *P* = 0.001). In men with predominant Gleason pattern 4, interestingly, the diabetes history was noted more frequently (8.4% versus 2.7%, *P* = 0.008). Accordingly, diabetes is involved in the incidence of high-grade/advanced prostate cancer most probably via the acquisition of more malignant potential under low-testosterone environment.

Meanwhile, the mechanism of lower PSA levels in diabetic men is hard to explain; it is still unclear why diabetes is associated with lower PSA levels. As described elsewhere, serum testosterone levels in men with type 2 diabetes are likely to be lower [22]. Yet, their deference from the normo-gonadotropic testosterone level is about 30% in median. It remains unknown whether the decrease of testosterone levels in such degree has an impact on serum PSA levels. Morgentaler advocated a theory that can account for such contradiction between androgen and PSA levels; there is a limit to the ability of testosterone to stimulate androgenic activities including prostate epithelium proliferation [30]. The Saturation Model explains the observation that prostate epithelium proliferation is testosterone dependent in serum testosterone concentrations at or below the near-castrate level (levels of 95% or more testosterone being deprived) and becomes testosterone-independent above this concentration. Physiologic concentrations of testosterone provide an excess of testosterone and its intracellular prostatic metabolite dihydrotestosterone, which maintains optimal prostatic growth. Reducing testosterone concentration below a critical concentration threshold (the Saturation Point) leads to an intracellular milieu where prostate tissue grows in an androgen-dependent manner [25, 30, 31]. Thus, the mild decrease of testosterone levels in diabetic men does not seemingly explicate their lower serum PSA level.

Another interest is whether a higher insulin level is associated with a higher incidence of prostate cancer. Stocks and colleagues prospectively performed conditional logistic regression analyses on 392 prostate cancer patients and 392 matched controls [32]. In their study, homeostatic model assessment of insulin resistance (HOMA-IR) was lower in the prostate cancer group than in the control group (1.5 ± 0.7 versus 1.6 ± 0.7), and the increasing level of HOMA-IR was associated with the decrease in risk of prostate cancer (Odds ratio = 0.60, 95% CI, 0.38–0.94, *P* = 0.03). In another case-control study by Chen et al. with 174 men in each of the case and control groups, insulin levels had no impact on the risk of incident prostate cancer [33]. In contrast, a recent cohort study with 9-year observation by Hammarsten and associates showed that the prediagnostic insulin level was higher in men with than without incident prostate cancer (fasting serum insulin 12.0 versus 9.0 mU/l, *P* = 0.023), although the study included a small number of prostate cancer patients (*n* = 44) and hazard ratio for the insulin level was unclear [34]. These varied results may possibly depend on study designs and length of the observation period. In a recent case-cohort study on a large registered cohort, Albanes et al. reported that increased insulin levels were associated with increased risks of prostate cancer (OR = 1.50–2.55 among compared insulin quartiles, *P* = 0.02) [35]. Another previous population-based study reported similar results [36].

In etiological approaches, the regulation and metabolism of insulin and IGF-1 are correlated, sharing homologous molecular structures [37], while many studies have shown the impact of high circulating IGF-1 levels on prostate carcinogenesis [38, 39]. This was also established experimentally before PSA era [40]. However, a recent large prospective study as well as previous studies has concluded the absence of correlation between the plasma IGF-1 level and insulin resistance [32]. In genetics, some reported no association between type 2 diabetes risk variants and prostate cancer risk [41], whereas some suggested a possible [42] or inverse [43] association between them.

Most recently, an experimental study reported intracellular *de novo* steroidogenesis promoted by insulin in prostate cancer; Lubik et al. showed that transcription of androgen-metabolic enzymes such as CYP17A1 and 5-*α*-reductase were upregulated by insulin in a dose-dependent manner in prostate cancer cells LNCaP and 22RV1, which express androgen receptor [44]. In their study, the protein level of CYP17A1 in LNCaP also increased significantly with insulin, and the intracellular level of dehydroepiandrosterone and testosterone increased 18-fold and 60-fold by insulin, respectively, (*P* < 0.05 in both) with PSA secretion increased significantly. These results suggest that insulin may directly promote proliferation of prostate cancer cells. However, these observations are based on an experimental model for castration-resistant prostate cancer, and studies to examine the effect of insulin on prostate tumorigenesis during its early phase or in hormone-naïve cancer are needed.

Thus, the relationship among insulin resistance, testosterone milieu, PSA level, incidence of prostate cancer, and its malignant potential in men with diabetes has not been fully elucidated, and remains a matter of concern for the regulation of prostate carcinogenesis as well as advances in management of prostate cancer in the general population. Further studies are required in both experimental and clinical approaches.

## 5. Practice of ADT

Both benign and malignant prostatic epithelial cells are well known to receive proliferative stimuli from androgens and to have androgen-dependent bioactivities, and ADT has been the therapeutic mainstay for men with metastasis or recurrent disease following definitive local therapy, although the treatment effect is palliative in most of the former [45]. ADT is performed with surgical castration or injection of gonadotropin-releasing hormone (GnRH) analogues with or without peroral antiandrogens. In 90s, the use of ADT rapidly increased from a small percent to 30% in the United States [46], and ADT has recently been used in about 30% of patients with localized or locally advanced prostate cancer, mainly combined with radiotherapy for intermediate- to high-risk disease [47]. It is estimated that more than 600,000 men receive ADT and that one in two prostate cancer patients experiences ADT in some treatment setting in the United States [48], whereas annual claims for GnRH analogues decreased by 25.1% and 16.8% from 2004 to 2007 in the Medicare and the Veterans Health Administration populations, respectively, most probably due to prevailing intermittent ADT and expectant management policy in increasing awareness about ADT-related adverse effects [49].

## 6. ADT-Related Insulin Resistance and Diabetes

It is estimated that the 5-year disease-specific survival for men with prostate cancer reaches 98% [3, 6]. In particular, men with localized prostate cancer almost exclusively die of other causes, and causes of death in them are similar to those of the general male population [50]. Therefore, ADT-related toxicity and the management thereof are critical in clinical practice.

As discussed elsewhere, reduced testosterone levels are associated with insulin resistance and type 2 diabetes in the general population [22]. Insulin resistance appears early during ADT; some previous prospective studies showed that increased fasting insulin levels already emerge in 26–63% of the patients 3 months after the inception of ADT [51, 52]. Hyperinsulinemia during the early period of ADT possibly counteracts against the development of diabetes. Yet, long-term ADT leads to a higher incidence of diabetes as shown in following large population-based studies, although there has been no prospective longitudinal study with a long observation period.

Keating et al. used Surveillance, Epidemiology, and End Results (SEER) Medicare data; the study cohort comprised 73,196 men with localized prostate cancer [53]. Among the 64,721 men without prevalent diabetes, 10.9% developed diabetes, and its adjusted hazard ratio was 1.44 (95% CI: 1.34 to 1.55, *P* < 0.001) in men treated with GnRH agonists. The same authors most recently performed another large population-based study and reported an increased risk of incident diabetes in men undergoing ADT with GnRH agonists (adjusted hazard ratio: 1.28, 95% CI: 1.19 to 1.38, *P* < 0.001) [2]. A Canadian population-based study also showed an increased incidence of diabetes in men treated with GnRH agonists (HR: 1.16, 95% CI: 1.11–1.21, *P* < 0.001) [54]. Another large study, though retrospectively, reported that 8.94% of men who were treated with ADT (*n* = 1,231) were diagnosed with diabetes 12 months after ADT, while 6.99% of those without ADT (*n* = 7,250) (*P* = 0.02) [55]. Thus, this evidence strongly supports the demand of large well-designed studies that longitudinally analyze the incidence of ADT-related metabolic disorders with long-term followup. Moreover, the pretreatment evaluation and posttreatment followup for diabetes and the relevant conditions are possibly important to improve overall survival in men receiving ADT. However, there has been no interventional study to determine appropriate/efficient screening methods and follow-up interval.

On the other hand, a few trials examined the effect of exercise, diet, and supportive agents/supplements in men during ADT. Nobes et al. reported the efficacy of a low glycemic index diet, exercise program, and metformin (850 mg daily to 850 mg twice daily) in men treated with GnRH agonist (6-month ADT) in a prospective randomized study [56]. The intervention arm (*n* = 20) had a reduction in abdominal girth (*P* = 0.05), weight (*P* < 0.001), and body mass index (*P* < 0.001) compared to controls (*n* = 20). Although the study was designed in a small pilot volume, changes in biochemical markers of insulin resistance did not differ between the two arms during the study. Lebret and colleagues examined the utility of an educational tool-kit consisting of information brochure concerning adverse effects of ADT, practical guidance on lifestyle, recipe booklet for ADT-adapted diet, and lifestyle diary to record and evaluate the life style and body measurement [57]. They recruited more than 500 men with prostate cancer receiving ADT, but the aim of the study was to test a tool-kit designed to improve well-being in patients with prostate cancer, and relevant studies on its impact on metabolic disorder during ADT are warranted.

The etiology of ADT-related diabetes is poorly understood. As mentioned above, increased fasting insulin levels are observed early after the initiation of ADT, suggesting possible primary responses to altered hormonal milieu. Additionally, recent studies showed that 6-month ADT with combined GnRH agonist and antiandrogens is associated with an about 10% increase of serum IGF-1 [58–61]. Although evidence supporting that low testosterone environment directly brings about the increased insulin level is absent, several previous studies have suggested associations among diabetes, cytokines, and sex steroid levels. Proinflammatory cytokines such as interleukin-6 (IL-6), and TNF-*α* secreted by macrophages and monocytes in response to infection play a critical role in immunity. Type 2 diabetes has been involved in innate immune system disorder with chronic low-grade inflammation [62, 63], and many studies have shown that serum/plasma levels of inflammatory markers represented by TNF-*α* and IL-6 in patients with elevated fasting blood glucose are independent values predictive of development of diabetes, thereby adipose tissue being the major source of these cytokines [64, 65]. Relevance of elevated serum IL-6, and TNF-*α* levels to insulin resistance and diabetes has been shown accordingly.

Besides, sex steroids such as 17beta-estradiol (estradiol) and testosterone have been suggested to play a role in modulating inflammation, although relevant studies are limited. A previous study showed that estradiol withdrawal brought about greater expressions of proinflammatory cytokines represented by IL-6 and TNF-*α* in human monocyte-derived macrophages of premenopausal women [66]. Concerning androgens, an *in vitro* study showed inhibition of IL-6 mRNA transcription and TNF release by dihydrotestosterone [67]. Some clinical trials have shown the influence of testosterone administration on cytokines or inflammation. In the double-blinded placebo-controlled crossover study on 20 hypogonadal type 2 diabetic men by Kapoor et al, although testosterone treatment reduced leptin (−7141.9 ± 1461.8 pg/mL; *P* = 0.0001) and adiponectin levels (−2075.8 ± 852.3 ng/mL; *P* = 0.02), its effect on the TNF-*α*, IL-6 or CRP level was not significant [68]; the small study volume may possibly lead to a negative result on cytokines.

Most recently, Kalinchenko and associates studied the effect of testosterone replacement on diabetic and inflammatory markers in 184 men with metabolic syndrome and hypogonadism in a randomized, placebo-controlled, double-blinded setting [69]. In the testosterone-treated group, plasma insulin and HOMA-IR decreased compared with those in the placebo-treated group (*P* = 0.07 and 0.04, resp.). Thereby, TNF-*α* and CRP of the testosterone-treated group declined 30 weeks after treatment compared to those at baseline (19 mg/dL versus 29 mg/L, *P* < 0.001 compared to control and 2.4 ng/l versus 3.5 ng/l, *P* = 0.03 compared to control, resp.); however, IL-6 levels were equivalent between before and after treatment (1.1 ng/l versus 1.1 ng/l). Additionally, subcutaneous abdominal fat has been shown to be an important index reflecting insulin resistance and relevant inflammation. A recent study focused on increased HOMA-IR (2.50 ± 1.12 to 2.79 ± 1.31, *P* < 0.05) and subcutaneous abdominal fat area 240.7 ± 107.5 to 271.3 ± 92.8 cm^2^, *P* < 0.01) [70], while abdominal fat mass has been associated with insulin resistance and the innate immune activation [71].

Regarding the effect of ADT on circulating proinflammatory cytokines, the relevant study has barely been presented. A recent study prospectively examined the relationship between these cytokines and sex steroid levels in the serum in 72 men with localized prostate cancer, who received ADT with GnRH agonists [72]. The authors reported an altered association of interleukin-6 with sex steroids during ADT as follows: before ADT, similar to the previous reports, serum interleukin-6 levels were inversely correlated with serum total-testosterone (Spearman's rank correlation coefficient rs = −0.305, *P* = 0.009) and dihydrotestosterone (rs = −0.308, *P* = 0.006) concentrations, but not correlated with adrenal androgen or estradiol levels. After ADT, in contrast to the pretreatment relationship, interleukin-6 levels were positively correlated with total-testosterone concentrations (rs = 0.343, *P* = 0.003), and were positively correlated also with levels of androstenedione (rs = 0.351, *P* = 0.002) and estradiol (rs = 0.335, *P* = 0.004), suggesting a coordinated regulation emerging between proinflammatory cytokines and sex steroids during ADT. Although the study focusing on ADT-related body composition change concluded the unchanged IL-6 level despite increased %body fat, the alteration of hormonal milieu produced by ADT can theoretically have an influence on the association of proinflammatory cytokines with metabolic activities including insulin sensitivity. However, it remains unclear whether such altered association between sex steroids and proinflammatory cytokines is the primary action with insulin resistance or secondary reaction to reduced testosterone levels, and further studies are warranted to elucidate the mechanism of ADT-related diabetes and to overcome this important adverse effect brought about by ADT.

## 7. Conclusion

Diabetes is associated with a lower PSA level in the general population and a higher incidence of advanced prostate cancer in the prostate cancer registration-based cohort. Although the mechanism of the former association is unknown, the latter can be explained by reduced testosterone levels in men with increased insulin resistance or type 2 diabetes. Insulin resistance is frequently observed early after the introduction of ADT, and long-term ADT links to the increased risk of development of diabetes. However, the mechanism of ADT-related diabetes remains unclear; a regulatory relationship between proinflammatory cytokines and sex steroids is possibly involved in ADT-related diabetes.

## Abbreviations

PSA: Prostate-specific antigen
ADT: Androgen deprivation therapy
GnRH: Gonadotropin-releasing hormone
HOMA-IR: Homeostatic model assessment of insulin resistance.
